# Body Composition and Abdominal Obesity in Patients With and Without Coronary Heart Disease

**DOI:** 10.14740/cr324w

**Published:** 2014-05-15

**Authors:** Aasghar Rahmani, Mohammadreza Hafezi Ahmadi, Samira Misgavam, Farhod Farhadi, Zahra Vahdat Shariatpanahi

**Affiliations:** a Department of Cardiology, Faculty of Medicine, Ilam University of Medical Sciences, Iran; b Department of Pathology, Faculty of Medicine, Ilam University of Medical Sciences, Iran; c National Nutrition and Food Technology Research Institute, Faculty of Nutrition and Food Technology, Shahid Beheshti University of Medical Sciences, Iran

**Keywords:** Body fat distribution, Stable angina, Fat mass, Waist circumference

## Abstract

**Background:**

The body fat and its distribution is an important risk factor for coronary artery diseases. The aim of this study was to evaluate the relationship between body composition and abdominal obesity in patients with and without coronary involvement in stable angina.

**Methods:**

One hundred and sixty-one patients who underwent coronary angiography for stable angina were divided into two groups: patients with or without coronary heart disease (CHD). Participants underwent bioimpedance analysis for measurement of adipose tissues and lean body mass.

**Results:**

No significant difference in body mass index and weight was found between two groups. Mean levels of waist circumference, waist to hip ratio and fat mass were significantly higher in CHD group (P = 0.02, P = 0.04 and P = 0.01). Fat-free mass was also significantly higher in non-CHD group (P = 0.02).

**Conclusions:**

Screening for adiposity in subjects by body composition measurement method and determining fat distribution could better identify those at higher risk for CHD.

## Introduction

Obesity is an expanding public health problem worldwide, creating a global health epidemic. Obesity has long been associated with an increased risk for coronary heart disease (CHD) [1]. Although the gold standard definition of obesity is considered an excess in body fat [2], the body mass index (BMI) is the most practical way to evaluate the degree of obesity. When estimating cardiovascular and other risks associated with obesity, regional fat distribution (waist circumference) and body composition must also be taken into account [3-5]. BMI gives no information about fat distribution and body fat content. Computed tomography scan and magnetic resonance imaging are accurate for measuring body composition but they are too expensive to be performed for this purpose alone. The bioimpedance analysis (BIA) is popular because it is safe, noninvasive, portable and rapid [6]. As BIA has been found to be a reliable measurement of body composition (fat-free mass and fat mass), this study was designed to evaluate the association of different indices of obesity in stable angina patients with and without CHD.

## Materials and Methods

In this cross-sectional study, we evaluated patients with stable angina who underwent coronary angiography in a teaching hospital. Stable angina pectoris refers to chest discomfort that occurs predictably and reproducibly at a certain level of exertion and is relieved with rest or nitroglycerin [7]. The study protocol was approved by responsible ethics committee and informed consents were obtained from all patients before performing the angiography. Exclusion criteria were unstable angina, myocardial infarction, fever, electrolyte imbalance, consumption of diuretics in the previous 24 h, corticosteroids and extreme obesity. Past medical history including diabetes, hypertension, dyslipidemia, tobacco use and demographic data was asked by a questionnaire. Diabetes was considered with a previously documented fasting glucose level of ≥ 126 mg/dL, a 2-h post-glucose challenge ≥ 200 mg/dL or treatment with antidiabetic medications. Hypertension was either self-reported or defined as a previously documented systolic blood pressure of ≥ 140 mmHg or a diastolic blood pressure of ≥ 90 mmHg or both. A previously documented total cholesterol of ≥ 200 mg/dL, triglyceride ≥ 150 mg/dL, LDL-C ≥ 100 mg/dL, HDL-C ≤ 40 mg/dL in males and ≤ 50 mg/dL in females was considered dyslipidemia. Coronary angiography was carried out via femoral artery with a standard method. The films of coronary angiography were reviewed by two cardiologists separately. According to the angiography reports, the patients were divided into two groups: patients with or without CHD. CHD was defined as stenosis more than 50% in angiography. Weight of participants was measured while the subjects were minimally clothed and bare feet using digital scales and recorded to the nearest 0.1 kg. Height was measured in a standing position and bare feet (without shoes) using a tape meter while the shoulders were in a normal state. BMI was calculated as weight (in kg) divided by the square of height (in meter). Overweight and obesity were defined as BMI > 25 kg/m^2^ and BMI > 30 kg/m^2^ consequently. Waist circumference was obtained by measuring the distance around the smallest area below the rib cage and above the umbilicus with the use of a non-stretch tape measure, without any pressure to body surface and measurements were recorded to the nearest 0.1 cm. A measurement of greater than 90 cm for men and greater than 80 cm for women was defined as abdominal obesity (the Caucasian criterion for abdominal obesity) [8]. Hip circumference was measured around the widest portion of the buttocks. Concomitantly all participants underwent whole-body bioelectrical impedance for the measurement of adipose tissues. Fat mass and fat-free mass were estimated from BIA data. Whole-body bioelectrical impedance was measured at 50 kHz and 800 MA with a bioimpedance meter (Maltron England). All measurements were done by the same investigator, using standard electrode positions. Statistical analyses SPSS software version 18 was used to analyze data. Student’s t test was used to compare continuous variables between the two groups. A value of P ≤ 0.05 was considered statistically significant.

## Results

A total of 161 patients with stable angina who underwent angiography were enrolled in the study. The mean ages of the patients with coronary involvement (n = 83) and without coronary involvement (n = 78) were 60.5 ± 18.8 and 58.7 ± 12.6 years, respectively (P = 0.8). Demographic data and past medical history of patients are shown in Table 1. There was no difference in the history of smoking between two groups (29% versus 22%; P = 0.6). The history of hypertension (47% versus 27%; P = 0.04) and dyslipidemia (48% versus 25%; P = 0.05) were significantly more in CHD group versus non-CHD group. The frequency of diabetes was also more in CHD group (22% versus 8%; P = 0.04). Table 2 lists the difference between anthropometric data and body composition in two groups. No significant difference in BMI, weight and hip circumference was found between CHD and non-CHD groups. The means of waist circumference, waist to hip ratio (WHR) and fat mass were significantly higher in CHD group. Fat-free mass was significantly higher in non-CHD group.

**Table 1:** Baseline Characteristics of the Subjects (Mean ± SEM)

	Coronary positive group (n = 83)	Coronary negative group (n = 78)	P value
Age	60.5 ± 18.8	58.7 ± 12.6	0.8
Sex (%)			
Male	43	41	0.8
Female	40	37	0.9
History of smoking	24	17	0.6
Diabetes	18	6	0.04
Dyslipidemia	40	20	0.05
Hypertension	39	21	0.04
Family history	20	10	0.04

**Table 2:** Anthropometric Data of the Subjects (Mean ± SEM)

	Coronary positive group (n = 83)	Coronary negative group (n = 78)	P value
Weight (kg)	74.47 ± 9.9	71.95 ± 11.7	0.1
BMI (kg/m^2^)	27.1 ± 3.4	26.1 ± 3.4	0.09
WC (cm)	104 ± 13.8	93 ± 13.2	0.02
HC (cm)	110 ± 9.8	105 ± 12.8	0.09
WHR	0.95 ± 0.09	0.88 ± 0.07	0.04
Fat mass (%)	30.59 ± 7.4	21.08 ± 7.1	0.01
Fat-free mass (%)	64.78 ± 8.6	71.48 ± 8.3	0.02

BMI: body mass index; WC: waist circumference; HC: hip circumference; WHR: waist to hip ratio

## Discussion

Our study showed that body fat content, abdominal obesity and WHR were significantly higher in CHD group versus non-CHD group and also there was no difference in mean levels of weight and BMI in two groups. The most practical way to evaluate the degree of obesity is BMI measurement, although it is not sensitive to body composition and fat distribution. Unfortunately BMI may overestimate the degree of obesity in individuals who are overweight but very muscular [9]. On the other hand, normal weight obesity, defined as the combination of normal BMI and high body fat content, is associated with increased risk for cardiovascular mortality [10]. Also older adults tend to have lower bone density and reduced lean body mass and therefore may weigh less than younger adults of the same height. Variation in body composition exists among different population groups as well as within the same group [11]. Blacks have greater bone mineral density and body protein as compared with whites [12]. In addition, BMIs for Asian populations need to be in the lower ranges for optimal health to reflect their higher cardiovascular risks [13]. Many other studies have shown that BMI does not reflect the actual body fat content, causing mistakes in the diagnosis of overweight or obesity [14-16].

Sadeghi et al showed that among anthropometrics and imaging indices of obesity, waist circumference and WHR have shown better association between central obesity with dyslipidemia in the patients with CHD, while computed tomography-measured visceral adipose tissue area has the best correlation with dyslipidemia in the patients without CHD [17]. Also Romero-Corral et al showed that BMI is not an independent CHD predictor and subjects with high body fat content detected by BIA with a normal BMI have a higher prevalence of cardiometabolic dysregulation and are at higher risk for cardiovascular mortality [10].

In our study, fat distribution was also significantly different in two groups, that is, central obesity was significantly more in CHD group. Indeed, waist circumference and WHR, as indicators of abdominal adiposity, have been shown to be better than BMI, an indicator of total adiposity, for identifying individuals at higher risk of developing atherosclerotic diseases [18, 19]. Recent studies have shown that when estimating cardiovascular and other risks associated with obesity, both regional fat distribution and comorbid conditions must also be taken into account. At any given level of BMI, the risk of the development of cardiovascular disease in both men and women is increased by more abdominal fat [20, 21]. As bioimpedance does not give any information about fat distribution, measuring of waist circumference and WHR beside BIA should be considered for assessment of cardiovascular risk factors. Our study has several potential limitations. First, it was a cross-sectional study. Second, given the small sample size in this trial, a detectable effect on outcome measures was not anticipated. Larger studies are needed to confirm these benefits.

### Conclusion

Differences in skeletal size and the proportion of lean body mass can contribute to body weight variations among individuals of similar height; therefore, body composition and fat distribution measurements should be used along with other assessment factors to provide an accurate description of one’s overall health.
